# Supplementary material for: Gas phase synthesis of [4]-helicene
Source: Nat Commun. 2019 Apr 3;10:1510. doi: 10.1038/s41467-019-09224-8 (PMC6447558; doi:10.1038/s41467-019-09224-8)
Supplement: Supplementary file 1 — Supplementary Information [file 41467_2019_9224_MOESM1_ESM.pdf]

Supplementary Information

Gas Phase Synthesis of [4]-Helicene

Zhao et al.

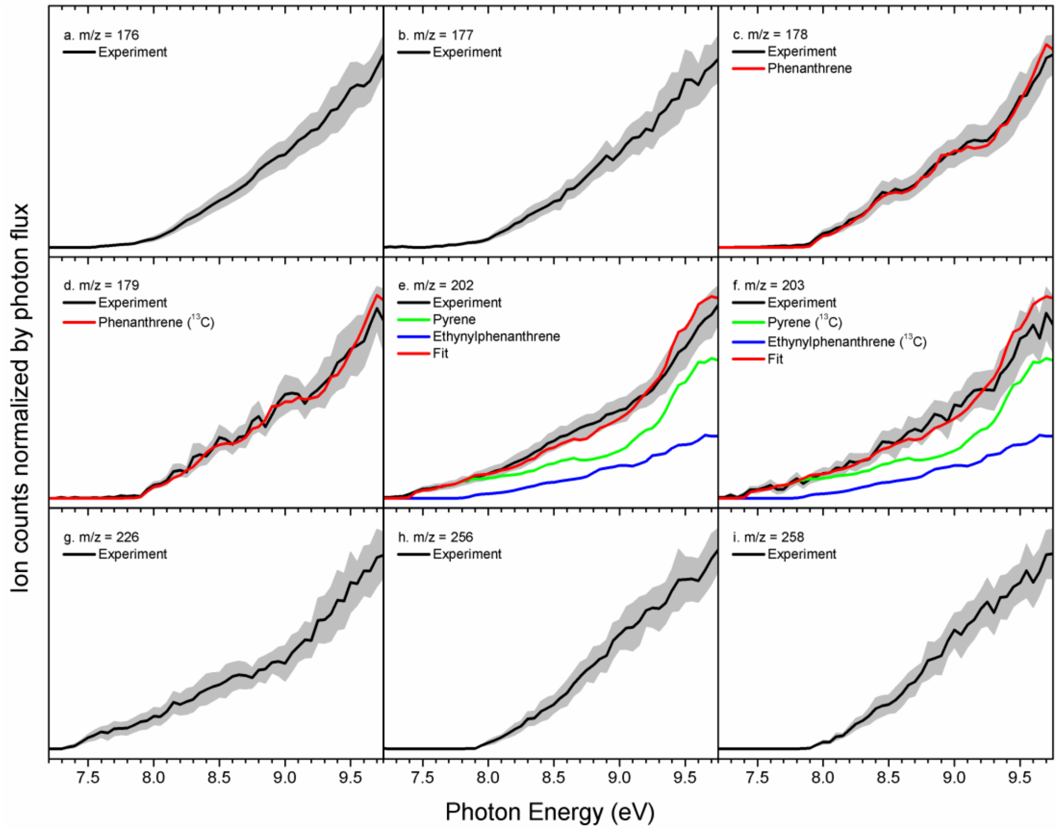

Supplementary Figure 1. PIE curves of distinct ions detected in the 4-phenanthryl - vinylacetylene system.

Signal at *m/z* = 202 and 203 can be associated with $C_{16}H_{10}$ molecule(s) and the ^{13}C-isotopially substituted counterpart(s) $^{13}CC_{15}H_{10}$, respectively. After scaling, both data sets are superimposable verifying that signal at *m/z* = 203 and 202 originate from the same (isotopically substituted) isomer. This signal can be fit with the PIE curves of pyrene and ethynylphenanthrene. Since the adiabatic ionization energies of distinct ethynylphenanthrene isomers are around 7.7 eV, and their PIE curves are similar,[1] the present work does not allow an identification of the specific ethynylphenanthrene isomer(s) formed. Further, at elevated temperatures, vinylacetylene can be pyrolyzed; acetylene can also be formed via the vinylacetylene plus atomic hydrogen reaction; overall, both pathways suggest that vinylacetylene transforms at a level of about 2% to acetylene. Acetylene can react with 4-phenanthrene via addition followed by hydrogen loss yielding ethynylphenanthrene.[1] Signal at *m/z* = 177 can be connected to the 4-phenanthrenyl radical. *m/z* = 176 and *m/z* = 178 originate from the hydrogen atom loss and hydrogen atom addition of 4-phenanthrenyl leading to phenanthryne isomers and phenanthrene, respectively. *m/z* = 178 and 179 can be both fit with the reference PIE curve of phenanthrene, verifying they are both attributed to phenanthrene with *m/z* = 179 resembling the ^{13}C-substituted phenanthrene.

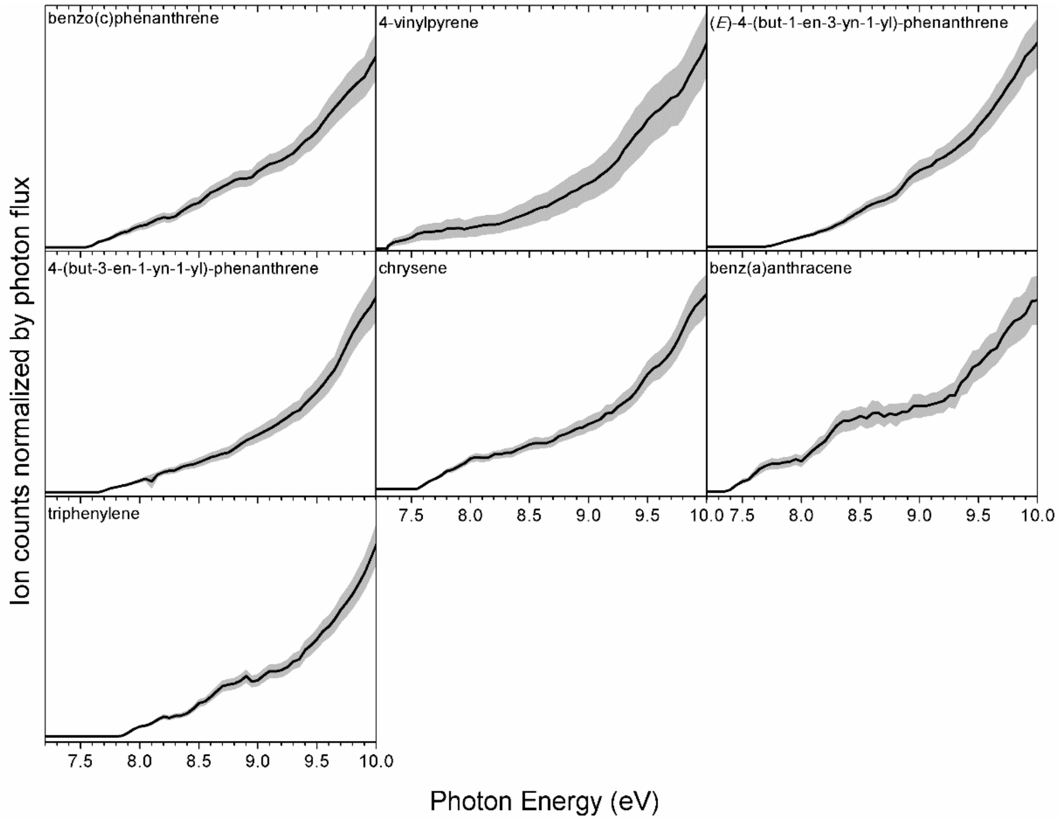

Supplementary Figure 2. PIE calibration curves for distinct $C_{18}H_{12}$ isomers: benzo(c)phenanthrene (4-helicene), 4-vinylpyrene, (*E*)-4-(but-1-en-3-yn-1-yl)-phenanthrene, 4-(but-3-en-1-yn-1-yl)-phenanthrene, chrysene, benz(a)anthracene, and triphenylene.

These PIE calibration curves of helium-seeded $C_{18}H_{12}$ isomers were newly recorded in this work under identical conditions (pressure, temperature) as the real experiments and are shown as black along with the error limits (grey area). The adiabatic ionization energies of these isomers are 7.55 ± 0.05 eV, 7.30 ± 0.05 eV, 7.70 ± 0.05 eV, 7.60 ± 0.05 eV, 7.55 ± 0.05 eV, 7.35 ± 0.05 eV, and 7.80 ± 0.05 eV, respectively. The literature values of photoionization energies for benzo(c)phenanthrene, chrysene, benz(a)anthracene, and triphenylene are 7.60 ± 0.02[2], 7.60 ± 0.01[3], 7.41 ± 0.02[2], and 7.84 ± 0.01[4]. Ionization energies for 4-vinylpyrene, (*E*)-4-(but-1-en-3-yn-1-yl)-phenanthrene, and 4-(but-3-en-1-yn-1-yl)-phenanthrene have not been reported prior to this work. The overall error bars consist of two parts: ±10% based on the accuracy of the photodiode and a 1 σ error of the PIE curve averaged over the individual scans.

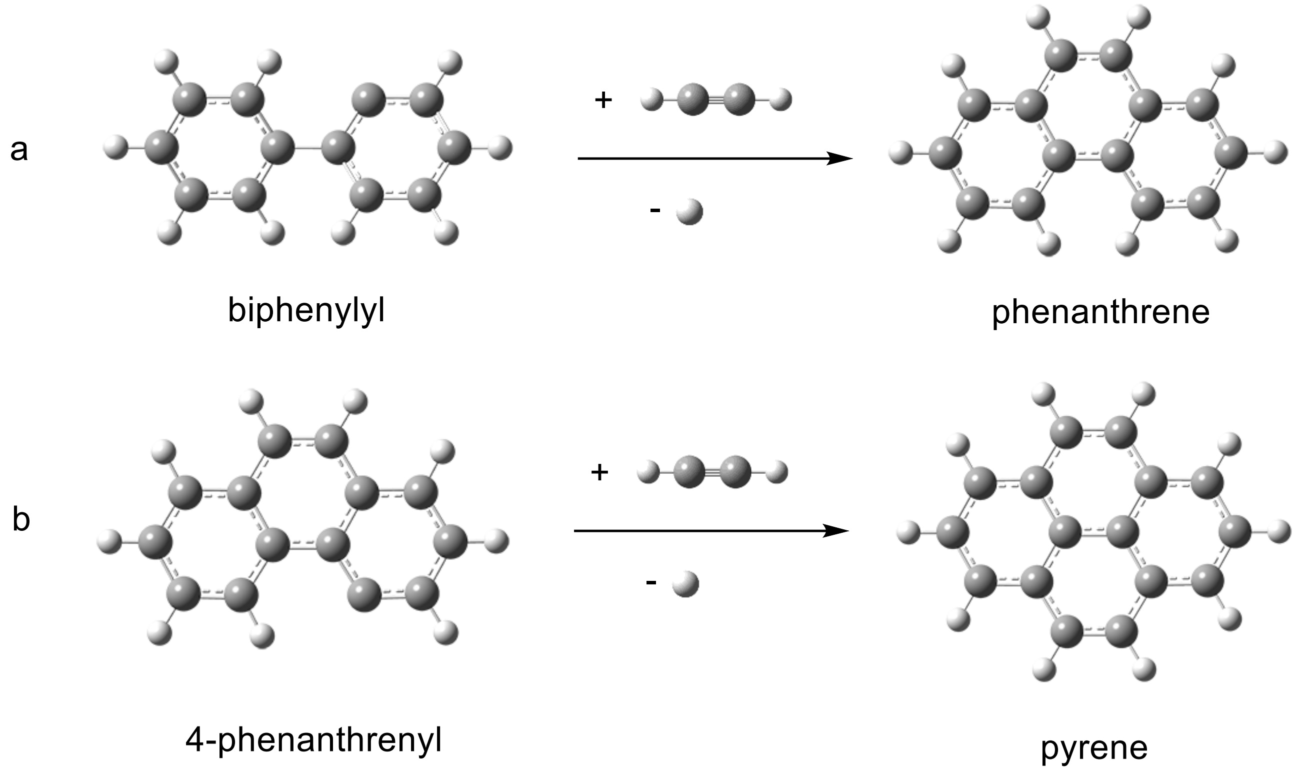

Supplementary Figure 3. a) Mechanism involving the reaction of biphenylyl ($[C_{12}H_9]^\bullet$) with acetylene (C_2H_2) to phenanthrene ($C_{14}H_{10}$) plus atomic hydrogen (H).[5] b) Mechanism involving the reaction of 4-phenanthrenyl ($[C_{14}H_9]^\bullet$) with acetylene (C_2H_2) to pyrene ($C_{16}H_{10}$) plus atomic hydrogen (H).[1]

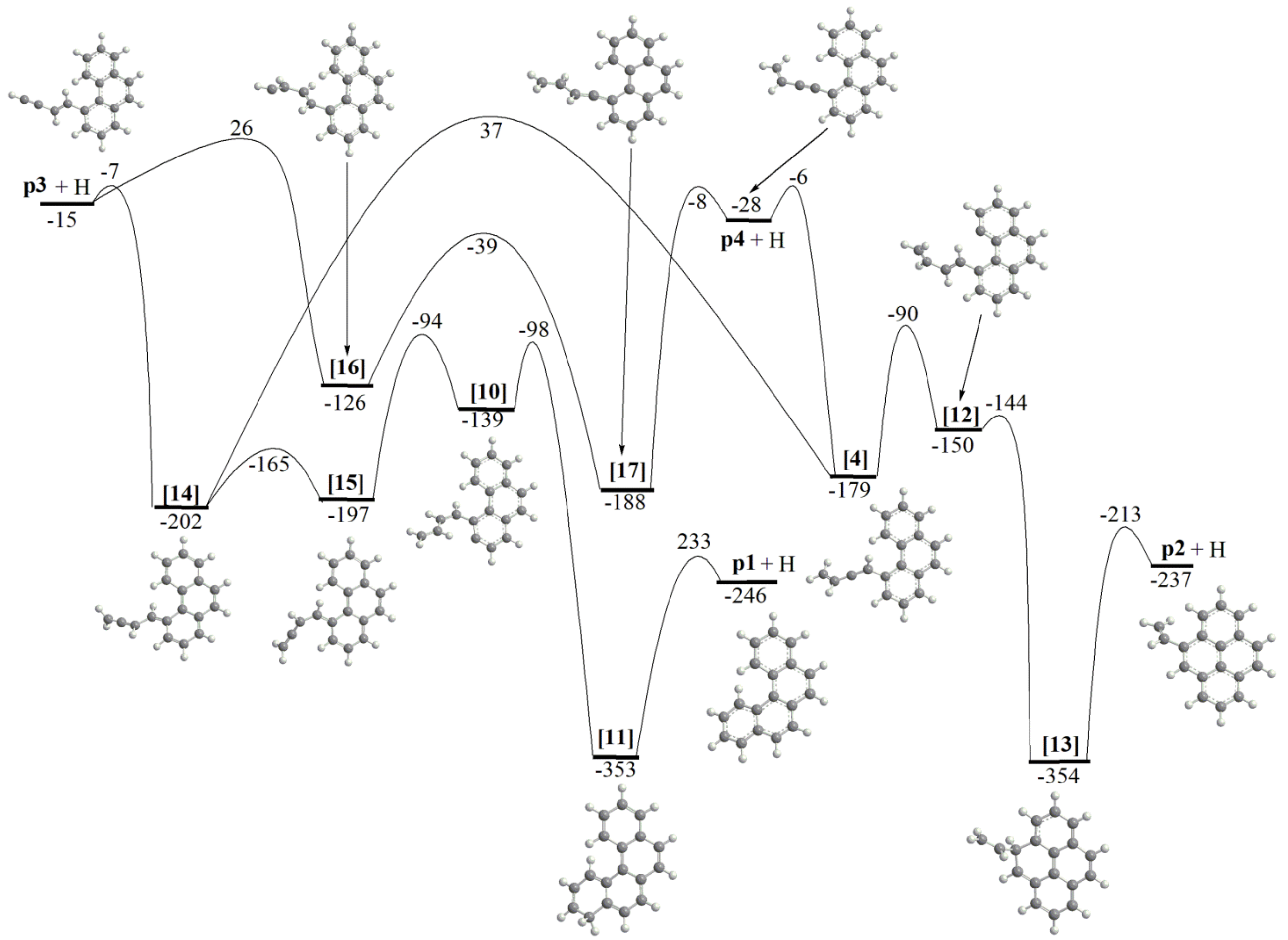

Supplementary Figure 4. PES for secondary, hydrogen atom assisted isomerization channels between the $C_{18}H_{12}$ products ([4]-helicene **p1**, 4-vinylpyrene **p2**, 4-((*E*)-but-1-en-3-yn-1-yl)phenanthrene **p3**, and 4-(but-3-en-1-yn-1-yl)phenanthrene **p4**) of the 4-phenanthrenyl $[C_{14}H_9]^{\bullet}$ reaction with vinylacetylene (C_4H_4) calculated at the G3(MP2,CC)//B3LYP/6-311G(d,p) level of theory. The relative energies are given in kJ mol^{-1}.

Supplementary Note 1: Rice-Ramsperger-Kassel-Marcus Master Equation (RRKM-ME) calculations of temperature- and pressure-dependent rate constants in the 4-phenanthrenyl plus vinylacetylene system

Phenomenological temperature- and pressure-dependent rate constants for the primary $C_{14}H_9$ + C_4H_4 and secondary $C_{18}H_{12}$ + H reactions were computed by solving the one-dimensional master equation[6] employing the MESS package.[7] Here, rate constants $k(T)$ for individual reaction steps were calculated within RRKM (unimolecular reactions) or transition state theory (TST, bimolecular reactions) generally utilizing the Rigid-Rotor, Harmonic-Oscillator (RRHO) model for the calculations of densities of states and partition functions for molecular complexes and the number of states for transition states. Collisional energy transfer rates in the master equation were expressed using the "exponential down" model,[8] with the temperature dependence of the range parameter α for the deactivating wing of the energy transfer function expressed as $\alpha(T) = \alpha_{300}(T/300\ \mathrm{K})^n$, with n = 0.62 and α_{300} = 424 cm^{-1} obtained earlier from classical trajectories calculations for the naphthyl radical ($C_{10}H_7$) plus argon system shown to be representative for acetylene addition reactions to C_8H_5 and C_8H_7 and also for vinylacetylene addition to C_6H_5 in argon or nitrogen bath gases.[9] We used collision parameters derived in the literature for similar systems; the Lennard-Jones parameters ε and σ for $C_{18}H_{13}$ intermediates were taken to be equal to those for pyrene put forward by Wang and Frenklach[10] and those for N_2 bath gas were taken from the works of Vishnyakov and co-workers.[11,12] The calculated rate constants for various reaction channels were then fitted to modified Arrhenius expressions $k = A{*}T^{\alpha}{*}\exp(-E_a/RT)$ or to a sum of two modified Arrhenius equations $k = A_1{*}T^{\alpha_1}{*}\exp(-E_a^1/RT) + A_2{*}T^{\alpha_2}{*}\exp(-E_a^2/RT)$, which are presented in the subsequent Section.

The RRKM-ME calculations of the rate constants have shown very little dependence on pressure at 0.01-0.1 atm and temperatures above 1000 K, i.e., under the conditions prevalent in the microreactor, and hence, single rate expressions were used in the subsequent simulations of the gas flow and chemical kinetics in the microreactor. According to the calculated rate constants, at the highest temperature in the microreactor, 1400 K, the branching ratios of the **p1**:**p2**:**p3**:**p4** are 1:169:6:51. This means that if only the primary $C_{14}H_9$ + C_4H_4 reaction occurs under these isothermal conditions, [4]-helicene **p1** is a minor product and 4-((*E*)-but-1-en-3-yn-1-yl)phenanthrene **p3** and 4-(but-3-en-1-yn-1-yl)phenanthrene **p4** should have been observed experimentally. However, as will be seen in the next section, the conditions in the microreactor are not isothermal and the residence time of 100-180 μs in the reactive zone where the pyrolysis of 4-bromophenanthrene and hence the primary $C_{14}H_9$ + C_4H_4 reaction can occur allows for secondary reactions to also take place. For instance, **p3** can add a hydrogen atom to the terminal carbon of the side chain to form **[14]**, which then undergoes *trans-cis*

conformational change to **[15]** followed by hydrogen migration from an aromatic ring to the side chain producing **[10]**, ring closure to **[11]**, and finally hydrogen loss forming [4]-helicene **p1**. The highest barrier on this pathway is as low as 8 kJ mol^{-1} for the initial hydrogen addition step, and the calculated rate constant is high, 1.2×10^{-10} cm^3 $molecule^{-1}$ s^{-1} at 1400 K. The reaction of **p4** with hydrogen predominantly produces the initial reactants and **p2** via the **p4** + H $\rightarrow$ **[4]** $\rightarrow$ **[2]** $\rightarrow$ $C_{14}H_9$ + C_4H_4 and **p4** + H $\rightarrow$ **[4]** $\rightarrow$ **[12]** $\rightarrow$ **[13]** $\rightarrow$ **p2** + H pathways, respectively, with the corresponding rate constants being 2.2×10^{-12} and 7.5×10^{-12} cm^3 $molecule^{-1}$ s^{-1} at 1400 K. These results indicate at the necessity of modeling of the gas flow in the microreactor together with kinetics, which takes into account most important primary and secondary reactions occurring inside the SiC tube.

Supplementary Note 2. Modeling of the gas flow and kinetics of the 4-phenanthrenyl – vinylacetylene system

In the modeling of the gas flow, we employed the COMSOL Multiphysics package and used the same axial symmetrical design model of the microreactor as described in the previous work on the phenyl – vinylacetylene system.[13] Specifications of the main details of the microreactor model, equations for the electric current, heat transfer, Navier-Stocks equation to describe gas motion in the silicon carbide tube, and mass transfer equations as well as physical properties of the materials exploited in the microreactor, boundary conditions for the heat transfer equations, for the Navier-Stocks equation, and for mass transfer equations - except for molar fractions of various gases at the inlet to the silicon carbide tube - were also identical to those described in the previous publication.[13] A gas mixture of C_4H_4 and He along with $C_{14}H_9Br$ was introduced as input gas at a temperature of $T = 323.0 \pm 0.5$ K upstream of the choke orifice at an inlet pressure $p = 300$ Torr upstream of the choke orifice. Since the vapor pressure of 4-bromophenanthrene is not known, its exact molar fraction in the molecular beam cannot be exactly evaluated and hence the simulations were carried out at various molar fractions of the constituents, as will be discussed below. The maximum temperature is 1400 ± 10 K at the silicon carbide microreactor surface. Diffusion coefficients for chemical species in helium were estimated using their diffusion coefficients in air which were taken from the handbook.[14] The list of chemical species involved and rate constants of all the reactions (the kinetic package) used are presented in Supplementary Tables 1 and 2. We implied the following kinetic mechanism, which included thermal unimolecular decomposition of $C_{14}H_9Br$ and C_4H_4, various product channels of the 4-phenanthrenyl ($C_{14}H_9$) + vinylacetylene (C_4H_4) reaction and H-assisted isomerization among its primary $C_{18}H_{12}$ products, as well as H-assisted decomposition of C_4H_4 and reactions of $C_{14}H_9$ with acetylene (C_2H_2) and H atoms. The rate constants were taken either from the RRKM-ME calculations described above or from the literature.

$C_{14}H_9Br \leftrightarrows C_{14}H_9 + Br$ (R1)

$C_4H_4 \rightarrow C_2H_2 + CCH_2/C_2H_2$ (R2)

$C_{14}H_9 + C_4H_4 \rightarrow$ [4]-helicene (**p1**) + H (R3a)

$C_{14}H_9 + C_4H_4 \leftrightarrows$ 4-((*E*)-but-1-en-3-ynyl)phenanthrene (**p3**) + H (R3b)

$C_{14}H_9 + C_4H_4 \leftrightarrows$ 4-(but-3-en-1-ynyl)phenanthrene (**p4**) + H (R3c)

$C_{14}H_9 + C_4H_4 \rightarrow$ 4-vinylpyrene (**p2**) + H (R3d)

4-((*E*)-but-1-en-3-yn1-1yl)phenanthrene (**p3**) + H $\rightarrow$ [4]-helicene (**p1**) + H (R4a)

4-((*E*)-but-1-en-3-yn-1-yl)phenanthrene (**p3**) + H $\leftrightarrows$

4-(but-3-en-1-yn-1-yl)phenanthrene (**p4**) + H (R4b)

4-((*E*)-but-1-en-3-yn-1-yl)phenanthrene (**p3**) + H → 4-vinylpyrene (**p2**) + H (R4c)

4-(but-3-en-1-yn-1-yl)phenanthrene (**p4**) + H → 4-vinylpyrene (**p2**) + H (R5a)

4-(but-3-en-1-yn-1-yl)phenanthrene (**p4**) + H → [4]-helicene (**p1**) + H (R5b)

$C_{14}H_9 + C_2H_2 \rightarrow C_{14}H_9CCH$ (4-ethynylphenanthrene) + H (R6a)

$C_{14}H_9 + C_2H_2 \rightarrow C_{16}H_{10}$ (pyrene) + H (R6b)

$C_4H_4 + H \leftrightarrows C_2H_2 + C_2H_3$ (R7)

$C_2H_3 \leftrightarrows C_2H_2 + H$ (R8)

$C_{14}H_9 + H \leftrightarrows C_{14}H_{10}$ (phenanthrene) (R9)

$C_{14}H_{10}$ (phenanthrene) + H $\leftrightarrows C_{14}H_9 + H_2$ (R10)

$C_{14}H_9 + C_4H_4 \leftrightarrows i\text{-}C_4H_3/n\text{-}C_4H_3 + C_{14}H_{10}$ (R11)

$H + C_4H_3 \leftrightarrows C_4H_4$ (R12)

$H + C_4H_4 \leftrightarrows H_2 + C_4H_3$ (R13)

In summary, these studies propose a **p1**:**p2**:**p3**:**p4** branching ratio of 1:169:6:51 at 1400 K indicating that [4]-helicene is only a minor product of the primary reaction of the 4-phenanthrenyl radical ($[C_{14}H_9]^\bullet$) with vinylacetylene (C_4H_4). This seems to be in contrast to our experimental findings. However, as described above **p3** can be converted to **p1** and **p4** can be converted to **p2** via secondary, hydrogen-assisted isomerization through facile pathways (Supplementary Figure 4). The simulations results show that within the error bars of the present RRKM-ME calculations and initial molar fractions of 4-bromophenanthrene and vinylacetylene in the molecular beam, we can achieve the **p1**:**p2**:**p3**:**p4** branching ratios to be 1:10:0.25:0.9, i.e., the yield of **p1** to exceed those of **p3** and **p4**. We should note at this point that the exact evaluation of the product branching ratios is not possible due to the fact that the absolute ionization cross sections are not known for any of the considered $C_{18}H_{12}$ isomers. For instance, if the absolute ionization cross section of **p1** significantly exceeds those of **p3** and **p4**, the contribution of the latter two isomers to the experimental PIE curve is masked, whereas that of [4]-helicene is enhanced. In the meantime, the simulations of the gas flow/kinetics clearly indicate that the three-ring-side-chain isomers **p3** and **p4** yield more stable four-ring PAHs [4]-helicene **p1** and 4-vinylpyrene **p2** via hydrogen-assisted isomerization and thus, the modeling results corroborate the experimentally observation that the 4-phenanthrenyl plus vinylacetylene reaction synthesizes [4]-helicene.

Supplementary Table 1. List of components involved in the reactions in the phenyl – vinylacetylene system.

List of species			
	Formula		Name
1	$C_{14}H_9Br$		4-Bromophenanthrene
2	$C_{14}H_9$		4-Phenanthrenyl radical
3	C_4H_4	CH_2=CHCCH	Vinylacetylene (But-1-en-3-yne)
4	C_2H_2	H-C≡C-H	Acetylene
5	$C_{18}H_{12}$	4 rings	**p1**, [4]-Helicene
6	$C_{18}H_{12}$	4 rings – C_2H_3	**p2**, 4-Vinylpyrene
7	$C_{18}H_{12}$	$C_{14}H_9$-CH=CH-C≡CH	**p3**, 4-((*E*)-but-1-en-3-yn-1-yl)phenanthrene
8	$C_{18}H_{12}$	$C_{14}H_9$-C≡C-CH=CH_2	**p4**, 4-(but-3-en-1-yn-1-yl)phenanthrene
9	H		Atomic hydrogen
10	$C_{16}H_{10}$	$C_{14}H_9$-C≡C-H	4-Ethynylphenanthrene
11	$C_{16}H_{10}$	4 rings	Pyrene
12	C_2H_3	CH_2=C-H	Vinyl radical
13	$C_{14}H_{10}$		Phenanthrene
14	H_2		Hydrogen molecule
15	Br		Bromine atom
16	C_4H_3		But-1-en-3-yn-2-yl

Supplementary Table 2. List of reactions and their nominal rate constants in the 4-phenanthrenyl – vinylacetylene system.

	Reaction		Rate constant, cm^6 mol^{-2} s^{-1}, cm^3 mol^{-1} s^{-1}, or s^{-1}	1,400 K cm^6 s^{-1}, cm^3 s^{-1}, s^{-1}
k_{1f}[a]	$C_{14}H_9Br \rightarrow C_{14}H_9 + Br$	0.01-0.1 atm	0.40881E+141×T^(-35.238)×exp(-87603/T) + 0.30916E+59×T^(-13.034)×exp(-49175/T)	543
k_{1r}[b]	$C_{14}H_9 + Br \rightarrow C_{14}H_9Br$	0.01-0.1 atm	0.20355E+47×6.02E+23×T^(-16.224)×exp(-19076/T)+0.24889E+07×6.02E+23*T^(-5.2016) ×exp(-2835.3/T)	$3.7 \cdot 10^{-11}$
$k_{2a}p2$[15]	$C_4H_4 \rightarrow C_2H_2 + CCH_2$	7.4 Torr	1.03e71×T^(-16.4)×exp(-121900/1.987/T) + 1.46e47×T^(-10.04)×exp(-98810/1.987/T)	3.84
$k_{2a}p3$[15]	$C_4H_4 \rightarrow C_2H_2 + CCH_2$	74 Torr	3.7e52×T^(-11.68)×exp(-102200/1.987/T) + 7.96e62×T^(-13.84)×exp(-118900/1.987/T)	7
k_{2a}	dependent on p		k_{2a}p2+(k_{2a}p3-k_{2a}p2)/log(10)×log(p/1333.3/7.4)	
$k_{2b}p2$[15]	$C_4H_4 \rightarrow C_2H_2 + C_2H_2$	7.4 Torr	4.22e68×T^(-16.04)×exp(-121600/1.987/T) + 3.04e43×T^(-9.31)×exp(-98770/1.987/T)	0.21
$k_{2b}p3$[15]	$C_4H_4 \rightarrow C_2H_2 + C_2H_2$	74 Torr	6.18e48×T^(-10.91)×exp(-101100/1.987/T) + 3.97e59×T^(-13.18)×exp(-118300/1.987/T)	0.5
k_{2b}	dependent on p		k_{2b}p2+(k_{2b}p3-k_{2b}p2)/log(10)×log(p/1333.3/7.4)	
k_2			$k_{2a} + k_{2b}$	
k_{3a}	$C_{14}H_9 + C_4H_4 \rightarrow$ [4]-helicene (**p1**) + H	0.01-0.1 atm	1.20E+97×T^(-24.89)×exp(-63197/1.987/T) + 1.32E+37×T^(-7.3076)×exp(35275/1.987/T)	$2.03 \cdot 10^{-15}$
k_{3bf}	$C_{14}H_9 + C_4H_4 \rightarrow$ **p3** + H	0.01-0.1 atm	1.50E+95×T^(-23.831)×exp(-67885/1.987/T) + 3.66E+22×T^(-2.6842)×exp(-29214/1.987/T)	$1.26 \cdot 10^{-14}$
k_{3br}	**p3** + H $\rightarrow C_{14}H_9 + C_4H_4$	0.01-0.1 atm	6.96E+104×T^(-25.896)×exp(-71708/1.987/T) + 7.11E+31×T^(-4.6443)×exp(-32797/1.987/T)	$4.68 \cdot 10^{-12}$
k_{3cf}	$C_{14}H_9 + C_4H_4 \rightarrow$ **p4** + H	0.01-0.1 atm	2.00E+58×T^(-12.501)×exp(-55014/1.987/T) + 1.63E+14×T^(-0.14926)×exp(-20294/1.987/T)	$1.02 \cdot 10^{-13}$
k_{3cr}	**p4** + H $\rightarrow C_{14}H_9 + C_4H_4$	0.01-0.1 atm	5.06E+67×T^(-14.72)×exp(-62072/1.987/T) + 6.80E+22×T^(-2.1359)×exp(-26925/1.987/T)	$2.18 \cdot 10^{-12}$
k_{3d}	$C_{14}H_9 + C_4H_4 \rightarrow$ **p2** + H	0.01-0.1 atm	4.33E+99×T^(-24.588)×exp(-74788/1.987/T) + 2.97E+28×T^(-4.7132)×exp(-15553/1.987/T)	$3.39 \cdot 10^{-13}$
k_{4a}	**p3** + H $\rightarrow$ [4]-helicene (**p1**) + H	0.01-0.1 atm	3.49E+50×T^(-10.732)×exp(-25927/1.987/T) + 3.65E-08×T^(5.7248)×exp(6429.8/1.987/T)	$1.22 \cdot 10^{-10}$
k_{4bf}	**p3** + H $\rightarrow$ **p4** + H	0.01-0.1 atm	8.68E+102×T^(-25.408)×exp(-69150/1.987/T) + 3.06E+40×T^(-7.1205)×exp(-36801/1.987/T)	$6.29 \cdot 10^{-12}$
k_{4br}	**p4** + H $\rightarrow$ **p3** + H	0.01-0.1 atm	7.78E+101×T^(-25.331)×exp(-71936/1.987/T) + 3.53E+39×T^(-7.0741)×exp(-39654/1.987/T)	$3.61 \cdot 10^{-13}$
k_{4c}	**p3** + H $\rightarrow$ **p2** + H	0.01-0.1 atm	1.18E+74×T^(-17.121)×exp(-57352/1.987/T) + 6.29E+47×T^(-9.5344)×exp(-46615/1.987/T)	$3.53 \cdot 10^{-13}$
k_{5a}	**p4** + H $\rightarrow$ **p2** + H	0.01-0.1 atm	4.33E+119×T^(-29.831)×exp(-89142/1.987/T) + 2.48E+36×T^(-6.6141)×exp(-19314/1.987/T)	$7.40 \cdot 10^{-12}$
k_{5b}	**p4** + H $\rightarrow$ **p1** + H	0.01-0.1 atm	3.26E+72×T^(-17.084)×exp(-58386/1.987/T) + 1.43E+43×T^(-8.5848)×exp(-45792/1.987/T)	$9.05 \cdot 10^{-15}$
k_{6a}[16]	$C_{14}H_9 + C_2H_2 \rightarrow$	0.01-0.1	1.17E+22×T^(-2.004)×exp(-40626/1.987/T) +	$6.70 \cdot 10^{-15}$

	$C_{14}H_9CCH$ + H	atm	1.7104×T^(3.7288)×exp(-18092/1.987/T)	
k_{6b}[16]	$C_{14}H_9 + C_2H_2 \rightarrow$ $C_{16}H_{10}$ (pyrene) + H	0.01-0.1 atm	8.08E+28×T^(-4.3076)×exp(-25873/1.987/T) + 2.80E+12×T^(1.92E-04)×exp(-7139.6/1.987/T)	$7.00\cdot10^{-13}$
k_7[17]	$C_4H_4+H \rightarrow C_2H_2+C_2H_3$		0.10023e17×T^(-0.47407)×exp(-12128/1.987/T)	$6.88\cdot10^{-12}$
k_{7r}[17]	$C_2H_2 + C_2H_3 \rightarrow H + C_4H_4$		0.11184e11×T^0.88224×exp(-6654.1/1.987/T)	$1.02\cdot10^{-12}$
k_8[18]	$C_2H_3 \rightarrow C_2H_2 + H$		3.94e12×(T/298)^1.62×exp(-155000/8.3/T)	$7.78\cdot10^{7}$
k_{8r}[19]	$C_2H_2 + H + He \rightarrow C_2H_3 + He$		3.31e-30×(6e23)^2×exp(-6150/8.31/T)	$1.95\cdot10^{-30}$
k_9[c]	$C_{14}H_9 + H \rightarrow C_{14}H_{10}$		exp(244.05-34871/T)×T^(-25.9)	$8.11\cdot10^{-11}$
k_{9r}p1[d]	$C_{14}H_{10} \rightarrow C_{14}H_9 + H$, p = 30 Torr		1.3489e108×T^(-25.81)×exp(-181750/1.987/T)	0.035
k_{9r}p2[d]	$C_{14}H_{10} \rightarrow C_{14}H_9 + H$, p = 50 Torr		6.3095e60×T^(-12.4)×exp(-148070/1.987/T)	0.047
k_{9r}[d]	$C_{14}H_{10} \rightarrow C_{14}H_9 + H$, 30<P<50		$k_{9r}p1+(k_{9r}p2-k_{9r}p1)/(50-30)\times(p/1333.3-30)$	
k_{10}[e]	$C_{14}H_{10} + H \rightarrow C_{14}H_9 + H_2$		1.22E+08×T^1.85×exp(-14800/1.987/T)	$6.57\cdot10^{-13}$
k_{10r}[f]	$C_{14}H_9 + H_2 \rightarrow C_{14}H_{10} + H$		1.07E+04×T^2.65×exp(-5556/1.987/T)	$5.26\cdot10^{-13}$
k_{11}[g]	$C_{14}H_9 + C_4H_4 \rightarrow$ *n,i*-C_4H_3 + $C_{14}H_{10}$		1.97e2×T^3.08×exp(-4463.1/1.987/T) + 3.41e2×T^3.11×exp(-8482.7/1.987/T)	$4.87\cdot10^{-13}$
k_{11r}[h]	*n,i*-$C_4H_3 + C_{14}H_{10} \rightarrow C_{14}H_9 + C_4H_4$		544.12×T^2.8952×exp(-14648/1.987/T) + 1936.1×T^3.0032×exp(-8387.1/1.987/T)	$4.5\cdot10^{-13}$
k_{12}p2[15]	$H + C_4H_3 \rightarrow C_4H_4$	7.5 Torr	8.16E+59×T^(-14.09)×exp(-20770.0/1.987/T) +5.88E+91×T^(-24.70)×exp(-26410.0/1.987/T)	$1.88\cdot10^{-12}$
k_{12}p3[15]	$H + C_4H_3 \rightarrow C_4H_4$	75 Torr	1.98E+65×T^(-15.12)×exp(-30460.0/1.987/T) +1.14E+56×T^(-13.08)×exp(-16980.0/1.987/T)	$3.66\cdot10^{-12}$
k_{12}	$H + C_4H_3 \rightarrow C_4H_4$	7.5<p<75	$(k_{16}p2+(k_{16}p3-k_{16}p2)/(75-7.5)\times(p/1333.3-7.5))$	
k_{12r}p2[15]	$C_4H_4 \rightarrow C_4H_3+H$	7.5 Torr	1.41E+65×T^(-14.94)×exp(-124900.0/1.987/T) + 2.19E+85×T^(-21.94)×exp(-125400.0/1.987/T)	0.04
k_{12r}p3[15]	$C_4H_4 \rightarrow C_4H_3 + H$	75 Torr	4.02E+69×T^(-15.72)×exp(-133600.0/1.987/T) + 3.48E+60×T^(-13.79)×exp(-119600.0/1.987/T)	0.22
k_{12r}[15]	$C_4H_4 \rightarrow C_4H_3 + H$	7.5<p<75	$(k_{16r}p2+(k_{16r}p3-k_{16r}p2)/(75-7.5)\times(p/1333.3-7.5))$	
k_{13}[i]	$H + C_4H_4 \rightarrow H_2 + C_4H_3$		4.47e8×T^1.88×exp(-14839/1.987/T)	$2.95\cdot10^{-12}$
k_{13r}[j]	$H_2 + C_4H_3 \rightarrow H + C_4H_4$		1.69e4×T^2.64×exp(-4559/1.987/T)	$1.10\cdot10^{-12}$

Notes: a. taken from 1-bromonaphthalene → 1-naphthyl + Br.[20]

b. taken from 1-naphthyl + Br → 1-bromonaphthalene.[20]

c. taken from $C_6H_5 + H \rightarrow C_6H_6$.[21]

d. taken from $C_6H_6 \rightarrow C_6H_5 + H$.[22]

e. Per site forward rate constant for H abstraction by H from an armchair PAH edge[23] multiplied by a factor of 2.

f. Reverse rate constant for H abstraction by H from an armchair PAH edge (from Ref. 23).[23]

g. taken from $C_6H_5 + C_4H_4 \rightarrow$ *n,i*-$C_4H_3 + C_6H_6$.[24]

h. taken from *n,i*-$C_4H_3 + C_6H_6 \rightarrow C_6H_5 + C_4H_4$.[24]

i. k_{13} is taken to be the same as for $C_6H_6 + H \rightarrow C_6H_5 + H_2$.[23]

j. k_{13r} is taken to be the same as for $C_6H_5 + H_2 \rightarrow C_6H_6 + H$.[23]

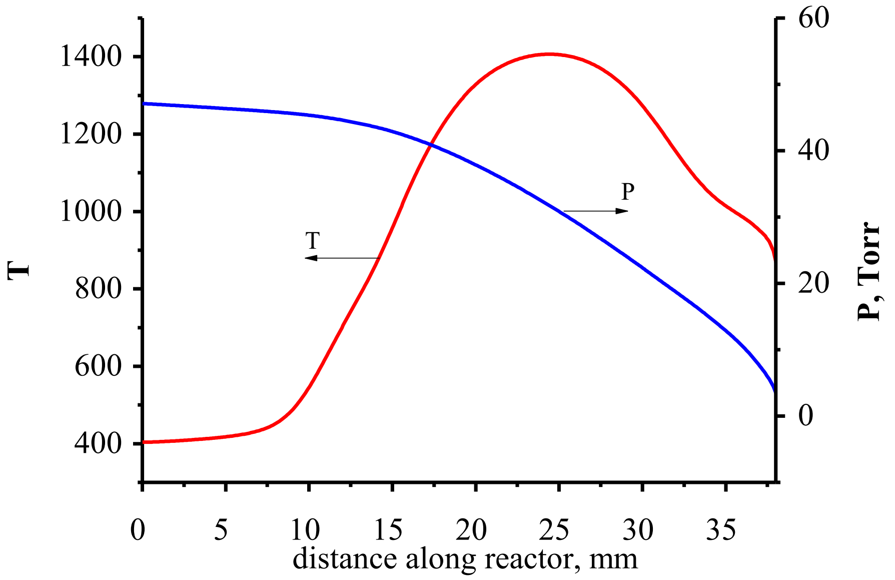

Supplementary Figure 5. Temperature (maximum 1400 K) and pressure distributions inside the microreactor silicon carbide tube.

Supplementary Figure 5 illustrates the calculated temperature and pressure distributions inside the microreactor. The product branching ratios **p1**:**p2**:**p3**:**p4** computed using nominal rate expressions presented in Supplementary Table 2 and estimated initial concentrations of the gases (0.1% $C_{14}H_9Br$, 5% C_4H_4, 95% He) are 1:158:5:35, which is close to the product branching ratios of the primary $C_{14}H_9$ + C_4H_4 reaction at 1400 K. This result means that the secondary H-assisted conversion of **p3** and **p4** to **p1** and **p2** does not have enough time to occur under these conditions. The rate of the secondary reactions can be increased at higher concentrations of the initial reactants, in particular, the 4-phenanthrenyl radical. With the nominal rate constant for $C_{14}H_9Br \rightarrow C_{14}H_9 + Br$ the decomposition efficiency of 4-bromophenanthrene, i.e., the fraction of $C_{14}H_9Br$ at the exit from the reactor in the blank experiment (i.e., without C_4H_4 present) at the same heating temperature of 1400 K, 14%, as compared to the experiment carried without heating the microreactor, is underestimated. Therefore, the rate constants for reaction (R1) were sampled within the error limits to reproduce the observed decomposition efficiency. This resulted in the **p1**:**p2**:**p3**:**p4** branching ratios of 1:114:4:29, which still cannot account for the experimental observation of only **p1** and **p2** and non-observation of **p3** and **p4**. We then probed the effects of several key rate constants for primary and secondary reactions in the $C_{14}H_9$ + C_4H_4 system (Supplementary Table 3) and also varied the initial concentrations of 4-bromophenanthrene and vinylacetylene.

The resulting branching ratios are shown in Supplementary Table 4. Branching ratios consistent with experiment can be obtained with initial 1% or 5% of $C_{14}H_9Br$ and 5% of C_4H_4 in the molecular beam

and even more so with 5% of $C_{14}H_9Br$ and 10% of C_4H_4. In the latter case, the calculated yield of **p1** exceeds those of **p3** and **p4**. A direct comparison between the calculations and experiment is complicated by the fact that the absolute ionization cross sections are not available for any of the considered $C_{18}H_{12}$ isomers. If the absolute ionization cross section of **p1** significantly exceeds those of **p3** and **p4**, the contribution of the latter two isomers to the experimental PIE curve is masked and can reside within the experimental error limits of the ion counts, whereas that of [4]-helicene is enhanced. Nevertheless, our simulations clearly indicate that the three-ring-side-chain isomers **p3** and **p4** yield to four-ring PAHs [4]-helicene **p1** and 4-vinylpyrene **p2** via H-assisted isomerization.

Supplementary Table 3. List of adjusted rate constants of the primary $C_{14}H_9$ + C_4H_4 and secondary $C_{18}H_{12}$ + H reactions used in the simulations. k_i(nom) are nominal rate constants.

	Reaction	forward	reverse
3a	$C_{14}H_9$ + C_4H_4 → **p1** + H	k_{3af}(nom) *3	0 (nom)
3b	$C_{14}H_9$ + C_4H_4 ⇆ **p3** + H	k_{3bf}(nom) *3	k_{3br}(nom)
3c	$C_{14}H_9$ + C_4H_4 ⇆ **p4** + H	k_{3cf}(nom) /3	k_{3cr}(nom)
3d	$C_{14}H_9$ + C_4H_4 → **p2** + H	k_{3df}(nom)	0 (nom)
4a	**p3** + H → **p1** + H	k_{4af}(nom) *3	0 (nom)
4b	**p3** + H ⇆ **p4** + H	k_{4bf}(nom) /3	k_{4br}(nom)*3
4c	**p3** + H → **p2** + H	k_{4cf}(nom) *3	0 (nom)
5a	**p4** + H → **p2** + H	k_{5af}(nom) *3	0 (nom)
5b	**p4** + H → **p1** + H	k_{5bf}(nom) *3	0 (nom)

Supplementary Table 4. Calculated branching ratios at various initial concentrations of 4-bromophenanthrene and vinylacetylene in the molecular beam.

Rate constant of reaction (R1)[a,b]	$C_{14}H_9Br$ molar fraction	C_4H_4 molar fraction	**p1**:**p2**:**p3**:**p4**
$(k_{1f})_1$, $(k_{1r})_1$	0.01	0.05	1 : 15.8 : 0.8 : 1.26
$(k_{1f})_1$, $(k_{1r})_1$	0.01	0.01	1 : 30 : 2.6 : 2.43
$(k_{1f})_2$, $(k_{1r})_1$	0.05	0.05	1 : 13.7 : 0.64 : 1.2
$(k_{1f})_2$, $(k_{1r})_1$	0.05	0.1	1 : 10 : 0.25 : 0.9
$(k_{1f})_3$, $(k_{1r})_3$	0.001	0.05	1 : 29 : 2.38 : 2.36

[a]$(k_{1f})_1$= (0.40881E+141*T^(-35.238)*exp(-82000/T)+0.30916E+59*T^(-13.034)*exp(-42000/T))
$(k_{1r})_1$=0.2*(0.40881E+141*T^(-35.238)*exp(-82000/T)+0.30916E+59*T^(-13.034)*exp(-42000/T))
$(k_{1f})_2$ =(0.40881E+141*T^(-35.238)*exp(-81000/T)+0.30916E+59*T^(-13.034)*exp(-41500/T))
$(k_{1f})_3$=0.1*(0.40881E+141*T^(-35.238)*exp(-85000/T)+0.30916E+59*T^(-13.034)*exp(-45000/T))
$(k_{1r})_3$=(0.20355E+47*6.02E+23*T^(-16.224)*exp(-19076/T)+0.24889E+07*6.02E+23*T^(-5.2016)*exp(-2835.3/T))/6

[b]Rate constants k_{1f} and k_{1r} were selected to reproduce about 14% of residual $C_{14}H_9Br$ (or about 86% of $C_{14}H_9Br$ pyrolysis) in the exit from the microreactor. The values of k_1 depend on the molar fractions of $C_{14}H_9Br$ and C_4H_4 because the residence time depends on their molar fractions.

Supplementary Note 3. Organic Synthesis of $C_{18}H_{12}$ isomers

General information

^{1}H (400 MHz) and ^{13}C (100.6 MHz) NMR spectra were recorded at ambient temperature in solution of $CDCl_3$. Reaction progress was monitored by TLC on Merck Kieselgel 60-F254 sheets with product detection by 254 nm light. Products were purified by column chromatography using Merck Kiselgel 60 (230-400 mesh). Reagent grade chemicals were used and solvents were dried by reflux and distillation from CaH_2 under N_2 unless otherwise specified.

Synthesis of 4-vinylpyrene; p2

The 4-vinylpyrene (**p2**), was synthesized by adopting Pd-catalyzed Stille cross-coupling reaction between 4-bromopyrene and Bu_3Sn(vinyl).

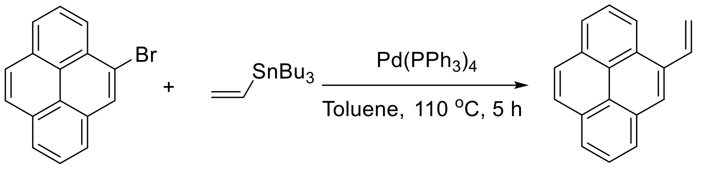

Supplementary Scheme 1. Synthesis of 4-vinylpyrene; **p2**

4-Vinylpyrene; p2

The 4-Bromopyrene (56.3 mg, 0.2 mmol) was dissolved in dry toluene in a flame-dried flask. Then $Pd(PPh_3)_4$ (11.6 mg, 0.01 mmol) and Bu_3Sn(vinyl) (64.3 µL, 69.8 mg, 0.22 mmol) were added at rt. The reaction mixture was heated at 110 °C and stirred for 5 h [progress of the reaction was monitored by TLC (hexane)]. Volatiles were evaporated and the residue was column chromatographed (n-hexane) to give **p2** (38 mg, 83%) as a white powder: ^{1}H NMR δ 5.63 (dd, J = 10.8, 1.6 Hz, 1H), 6.02 (dd, J = 17.2, 1.6 Hz, 1H), 7.65 (dd, J = 17.2, 11.2 Hz, 1H), 7.99-8.09 (m, 4H), 8.16-8.21 (m, 4H), 8.43 (d, J = 7.6 Hz, 1H); ^{13}C NMR δ 117.81, 121.72, 124.52, 124.85, 124.91, 125.09, 125.39, 125.91, 126.18, 127.41, 127.75, 129.99, 131.10, 131.17, 131.59, 135.09, 135.22, 135.45.

Synthesis of (*E*)-4-(but-1-en-3-yn-1-yl)phenanthrene; p3

The (*E*)-4-(but-1-en-3-yn-1-yl)-phenanthrene, **p3** was synthesized by stereoselective conversion of 4-ethynylphenanthrene into *trans*-1-alkenyliodide **A** via hydroboration-hydrolysis-iodination sequence adopted from literature[25] (**Supplementary Scheme 2**). Thus, treatment of 4-ethynylphenanthrene (**E**, **Supplementary Scheme 3**) with catecholborane at 70 °C gave intermediary boronic ester, which was hydrolyzed with H_2O to give *trans*-alkenylboronic acid, **A** in 37% yield after purification on silica gel column. Treatment of the purified (catechol free) **A** in ether solution with iodine (1.2 equiv) in the presence of aqueous NaOH (3.0 equiv) at 0 °C provided alkenyliodide **B** (50%). Subsequent Sonogashira coupling of **B** with (trimethylsilyl)acetylene yielded **C** (85%), which on desilylation with K_2CO_3 in MeOH/DCM gave (*E*)-4-(but-1-en-3-yn-1-yl)-phenanthrene, **p3** (92%).

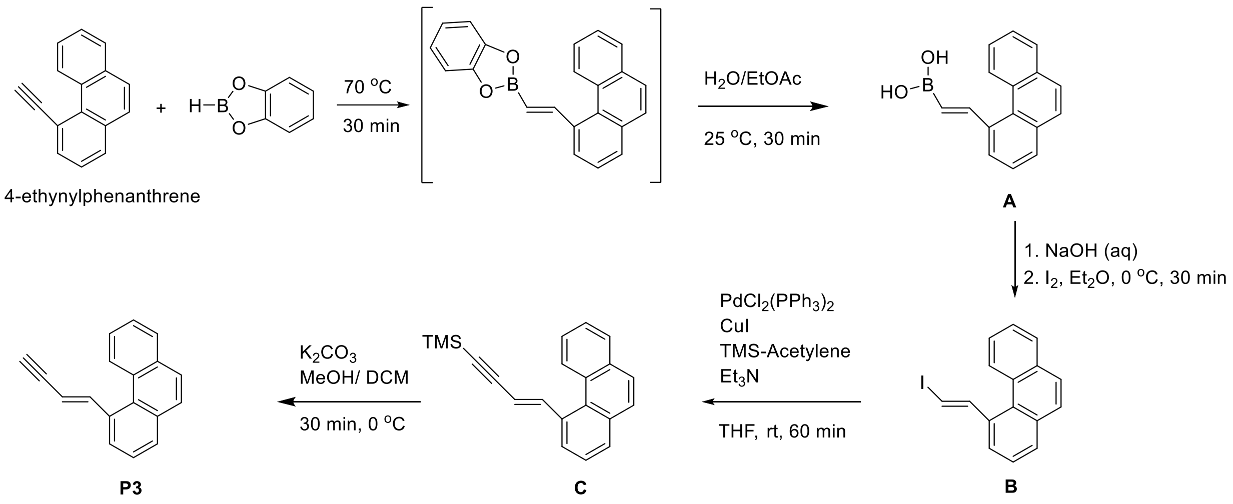

Supplementary Scheme 2. Synthesis of (*E*)-4-(but-1-en-3-yn-1-yl)-phenanthrene; **p3**

(*E*)-4-(2-iodovinyl)phenanthrene; B.

Step a. The 4-ethynylphenanthrene (101.1 mg, 0.5 mmol) and catecholborane (53.2 µL, 60 mg, 0.5 mmol) were placed in a flame-dried flask under N_2 at ambient temperature and the reaction mixture were stirred for 60 min at 70 °C. Then H_2O/EtOAc (1:1; 10 mL) were added and stirring was continued for 30 min at 25 °C to effect the hydrolysis of boronic ester. The reaction mixture was extracted with EtOAc, organic layer separated and the aqueous layer was back extracted with EtOAc twice. The combined organic layer was dried (Na_2SO_4) and evaporated. The residue was column chromatographed (20-40% EtOAc in hexane) to give **A** (45 mg, 37%) as a gummy solid, which was directly used for the next step. *Step b.* The boronic acid **A** (45 mg, 0.18 mmol) was dissolved in 5 mL Et_2O in a 25 mL flask and cooled to 0 °C. Then aqueous NaOH (180 µL, 3 N, 0.54 mmol) was added dropwise followed by elemental iodine (54.8 mg, 0.22 mmol) dissolved in 5 mL Et_2O, while stirring at 0 °C. The reaction mixture was stirred for 30 min at 0 °C. The excess I_2 was destroyed by addition of few drops of aqueous $Na_2S_2O_3$ solution. The reaction mixture was extracted with Et_2O and the organic layer was separated and the aqueous layer was back extracted with Et_2O twice. The combined organic layer was dried (Na_2SO_4) and evaporated. The residue was column chromatographed (*n*-hexane) to give **B** (30 mg, 50%) as a white powder: ^{1}H NMR δ 6.85 (d, J = 14.8 Hz, 1H), 7.53-7.59 (m, 2H), 7.62-7.70 (m, 2H), 7.72-7.77 (m, 2H), 7.87-7.94 (m, 2H), 8.17 (d, J = 14.8 Hz, 1H), 8.72 (d, J = 8.4 Hz, 1H); ^{13}C NMR δ 125.67, 126.24, 126.27, 126.62, 127.35, 127.78, 127.98, 128.16, 128.73,128.74, 129.33, 130.62, 133.36, 133.54, 137.36, 149.46.

(*E*)-Trimethyl(4-(phenanthren-4-yl)but-3-en-1-yn-yl)silane; C.

$Pd(PPh_3)_2Cl_2$ (4.2 mg, 0.006 mmol) and Cu(I)I (2.3 mg, 0.012 mmol) were added to dry THF (2 mL) in a flame-dried round bottom flask equipped with a stir bar under N_2 at 0 °C (ice-bath). Then iodovinylphenanthrene, **B** (30 mg, 0.09 mmol) was added followed by TMS-acetylene (18.6 µL, 13.2 mg, 0.14 mmol) and Et_3N (25.2 µL, 18.3 mg, 0.18 mmol). The resulting mixture was allowed to warm up to ambient temperature and was stirred for 1h [progress of the reaction was monitored by TLC (*n*-hexane)]. Volatiles were evaporated and the residue was column chromatographed (*n*-hexane) to give **C** as a light yellow gummy solid (23 mg, 85%): ^{1}H NMR δ 0.30 (s, 9H), 6.28 (d, J = 16.0 Hz,

1H), 7.53-7.69 (m, 4H), 7.71-7.79 (m, 3H), 7.83-7.87 (m, 1H), 7.91-7.94 (m, 1H), 8.72 (d, J = 8.4 Hz, 1H); ^{13}C NMR δ 0.20, 96.30, 104.73, 109.08, 126.11, 126.22, 126.58, 127.33, 127.66, 128.31, 128.53, 128.57, 129.34, 130.76, 133.35, 133.52, 135.67, 139.39, 146.46.

(*E*)-4-(But-1-en-3-yn-1-yl)phenanthrene; p3.

Anhydrous K_2CO_3 (12 mg, 0.09 mmol) was added to a stirred solution of **C** (23 mg, 0.08 mmol) in 2 mL MeOH/DCM (1:1) at room temperature. After for 30 min, volatiles were evaporated and the residue was column chromatographed (*n*-hexane) to give **p3** (16 mg, 92%) as a clear liquid: ^{1}H NMR δ 3.13 (d, J = 2.0 Hz, 1H), 6.23 (dd, J = 16.0, 2.4 Hz, 1H), 7.56-7.69 (m, 4H), 7.72-7.77 (m, 2H), 7.85-7.89 (m, 2H), 7.91-7.94 (m, 1H), 8.67 (d, J = 7.6 Hz, 1H); ^{13}C NMR δ 78.75, 83.24, 107.98, 126.13, 126.23, 126.64, 127.34, 127.71, 128.30, 128.41, 128.62, 128.91, 129.48, 130.72, 133.38, 133.55, 135.42, 147.43.

Synthesis of 4-(but-3-en-1-yn-1-yl)phenanthrene; p4

For the synthesis of 4-(but-3-en-1-yn-1-yl)-phenanthrene **p4**, 4-bromophenanthrene[1] was converted to TMS protected 4-ethynylphenanthrene **D** (**Supplementary Scheme 3**) by CuI/Pd(PPh_3)$_4$-mediated Sonogashira coupling with (trimethylsilyl)acetylene. Desilylation of **D** with K_2CO_3 gave 4-ethynylphenanthrene **E**. Subsequent Sonogashira coupling between **E** and vinyl bromide resulted in the formation of 4-(but-3-en-1-yn-1-yl)-phenanthrene **p4**.

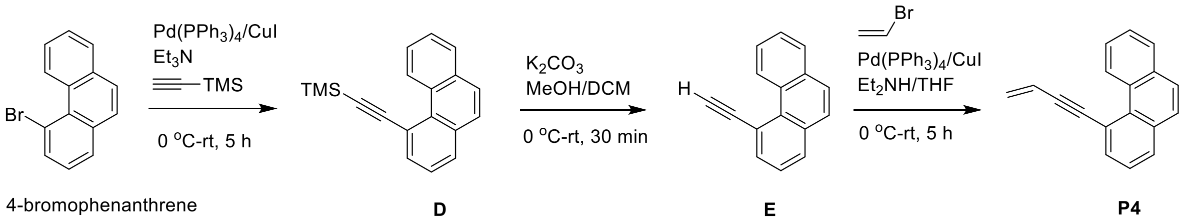

Supplementary Scheme 3. Synthesis of 4-(but-3-en-1-yn-1-yl)-phenanthrene; **p4**

4-[2-(Trimethylsilyl)ethynyl]phenanthrene; D.

Pd(PPh_3)$_4$ (43.3 mg, 0.037 mmol) and Cu(I)I (6.9 mg, 0.036 mmol) were added to dry Et_3N (10 mL) flame-dried round bottom flask equipped with a stir bar. Then 4-bromophenanthrene (300 mg, 1.17 mmol) was added followed by TMS-acetylene (292 µL, 207 mg, 2.11 mmol). The resulting mixture was stirred at ambient temperature for 5 h [progress of the reaction was monitored by TLC (hexane)]. The reaction mixture was then diluted with dichloromethane (DCM) and filtered through a short pad of silica. Volatiles were evaporated and the residue was column chromatographed (n-hexane) to give trimethyl(phenanthren-4-ylethynyl)silane **D** as a light yellow solid (257 mg, 80%): ^{1}H NMR δ 0.39 (s, 9H), 7.52 (t, J = 7.6 Hz, 1H), 7.61-7.66 (m, 2H), 7.67-7.76 (m, 2H), 7.86-7.93 (m, 3H), 10.43-10.47 (m, 1H); ^{13}C NMR δ 0.04, 100.80, 108.23, 119.34, 125.56, 125.67, 126.65, 127.08, 127.48, 128.03, 128.45, 130.11, 130.13, 130.83, 133.16, 133.18, 135.62.

4-Ethynylphenanthrene; E.

Anhydrous K_2CO_3 (250 mg, 1.8 mmol) was added to a stirred solution of **D** (412 mg, 1.5 mmol) in 10 mL MeOH/DCM (1:1) at room temperature. After for 30 min, volatiles were evaporated and the residue was column chromatographed (*n*-hexane) to give **E** (249 mg, 82%) as a light yellow solid: ^{1}H NMR δ 3.70 (s, 1H), 7.54 (t, *J* = 7.8 Hz, 1H), 7.63-7.68 (m, 2H), 7.69-7.78 (m, 2H), 7.89-7.97 (m, 3H), 10.34-10.38 (m, 1H); ^{13}C NMR δ 83.59, 86.54, 118.29, 125.63, 126.09, 126.35, 127.19, 127.42, 128.13, 128.55, 130.26, 130.42, 130.69, 133.15, 133.22, 136.27.

4-(But-3-en-1-yn-1-yl)phenanthrene; p4.

$Pd(PPh_3)_4$ (8.1 mg, 0.007 mmol) and Cu(I)I (5.3 mg, 0.028 mmol) were placed in the flame-dried flask under N_2 at 0 ^{o}C (ice-bath). Then Et_2NH (0.90 mL, 636 mg, 8.70 mmol) and vinyl bromide (1.0 M in THF; 0.90 mL, 0.90 mmol) were added. Next, 4-ethynylphenanthrene **E** (140. mg, 0.69 mmol) dissolved in dry THF (2 mL) was added slowly via a syringe pump (over 3 h) and the resulting mixture was allowed to warm up to ambient temperature (30 min) and was stirred for another 2 h. Volatiles were evaporated and residue was column chromatographed (*n*-hexane) to give **p4** [13 mg, 8.3%; TLC (hexane) R_f = 0.6] as a clear liquid. ^{1}H NMR : δ 5.63 (dd, *J* = 11.2, 2.0 Hz, 1H), 5.89 (dd, *J* = 17.2, 2.0 Hz, 1H), 6.25 (dd, *J* = 17.6, 11.2 Hz, 1H), 7.53 (t, *J* = 7.6 Hz, 1H), 7.62-7.69 (m, 2H), 7.70-7.77 (m, 2H), 7.87-791 (m, 3H), 10.23-10.25 (m, 1H); ^{13}C NMR : δ 92.96, 94.00, 117.79, 119.36, 125.67, 126.12, 126.33, 126.99, 127.04, 127.48, 128.03, 128.53, 129.92, 130.84, 133.19, 133.28, 135.07.

Note. Also isolated from the reaction mixture was a dimer resulting from homocoupling of **E** TLC (hexane) R_f = 0.3].

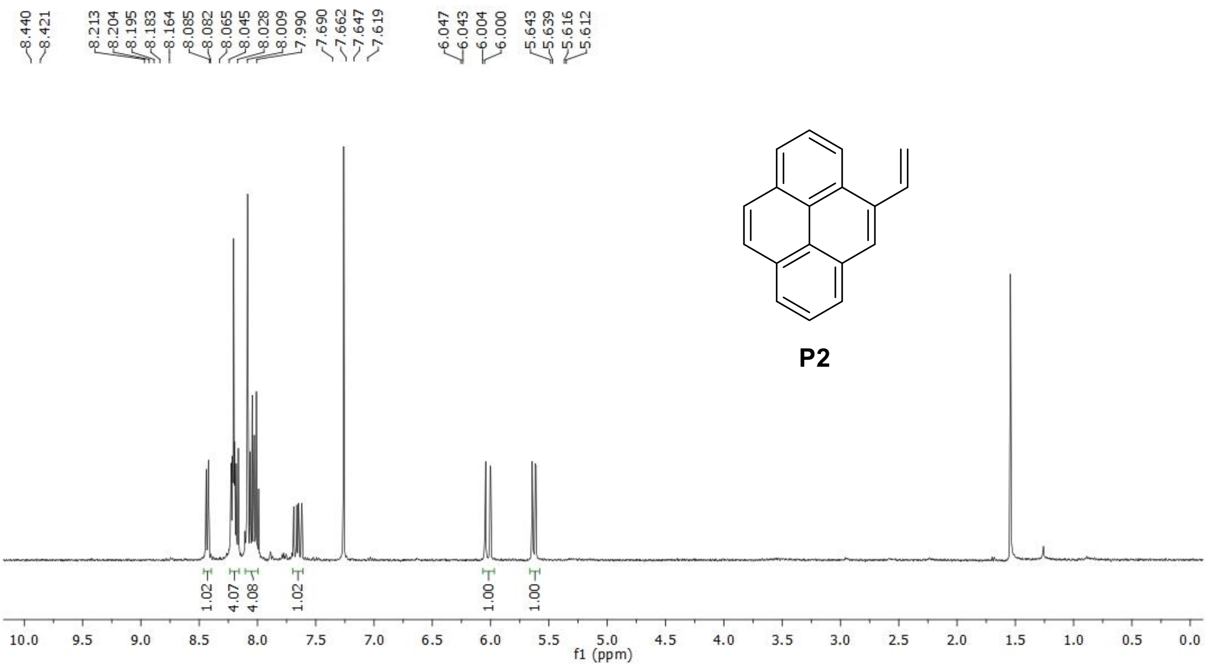

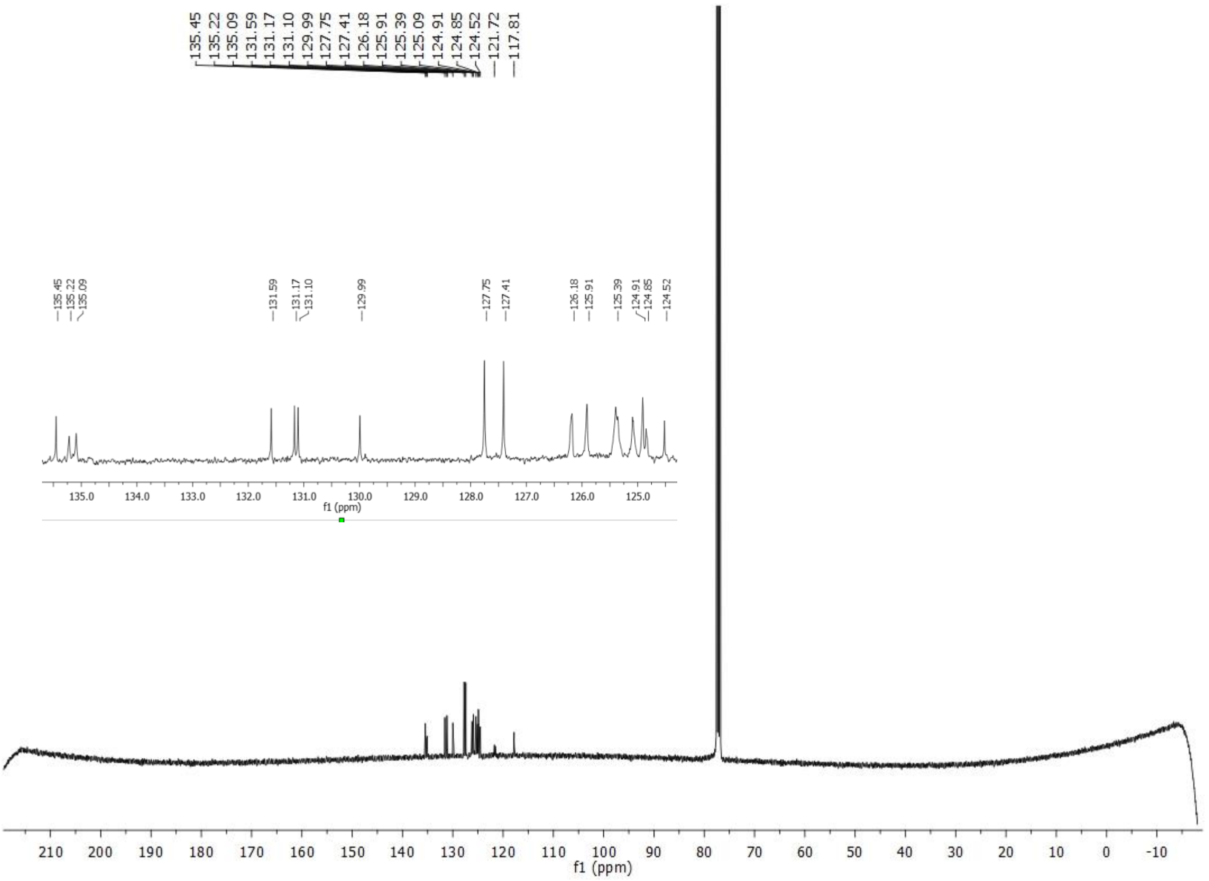

Supplementary Figure 6. ^{1}H NMR and ^{13}C NMR spectra of compound **p2**.

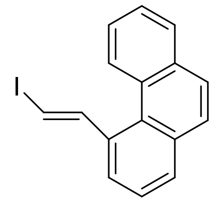

B

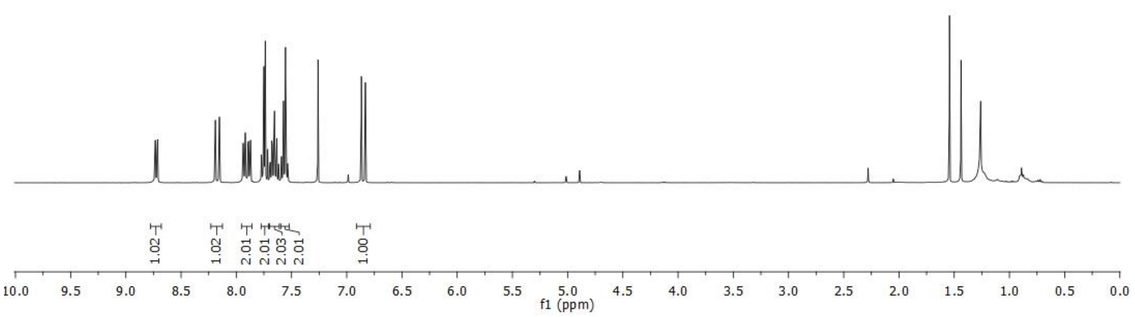

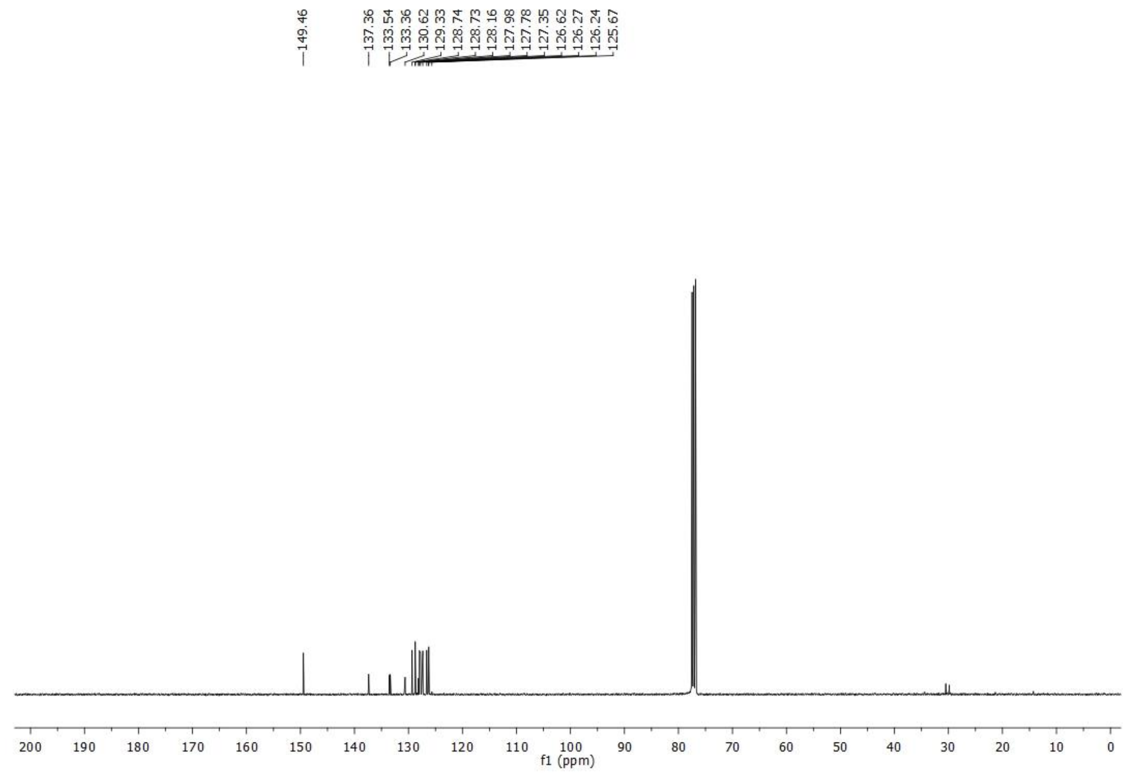

Supplementary Figure 7. ^{1}H NMR and ^{13}C NMR spectra of compound **B**.

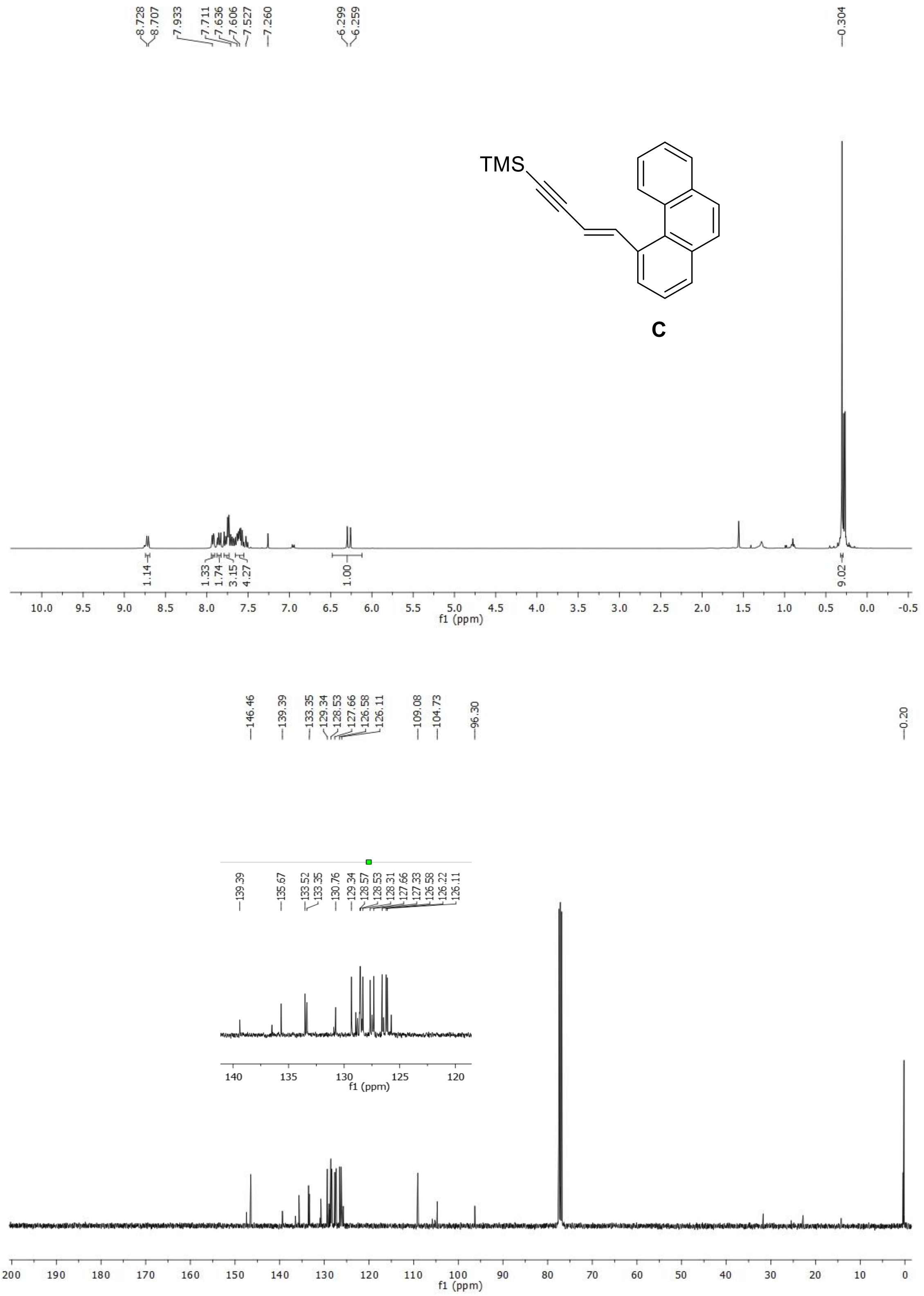

Supplementary Figure 8. ^{1}H NMR and ^{13}C NMR spectra of compound **C**.

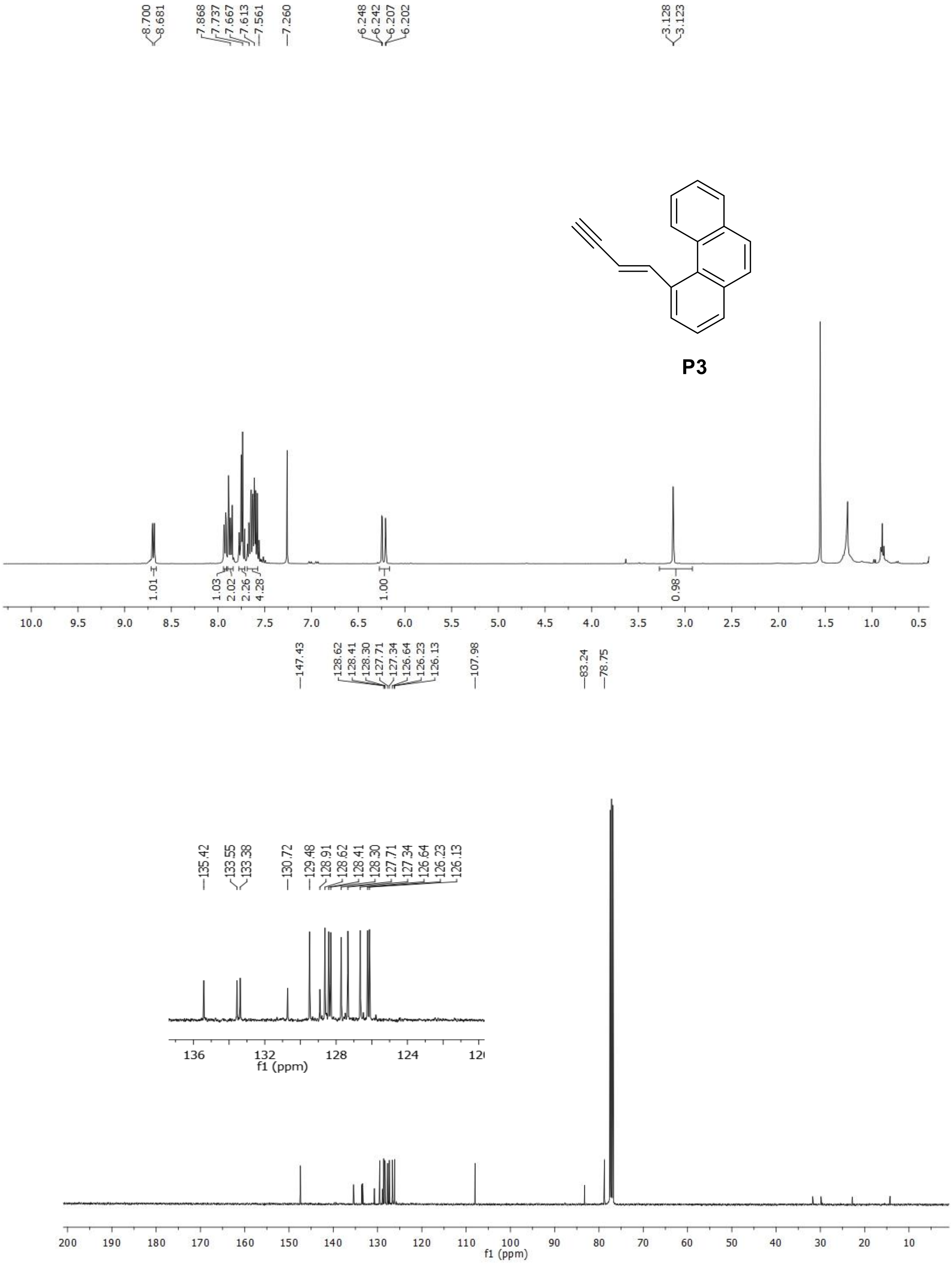

Supplementary Figure 9. ^{1}H NMR and ^{13}C NMR spectra of compound **p3**.

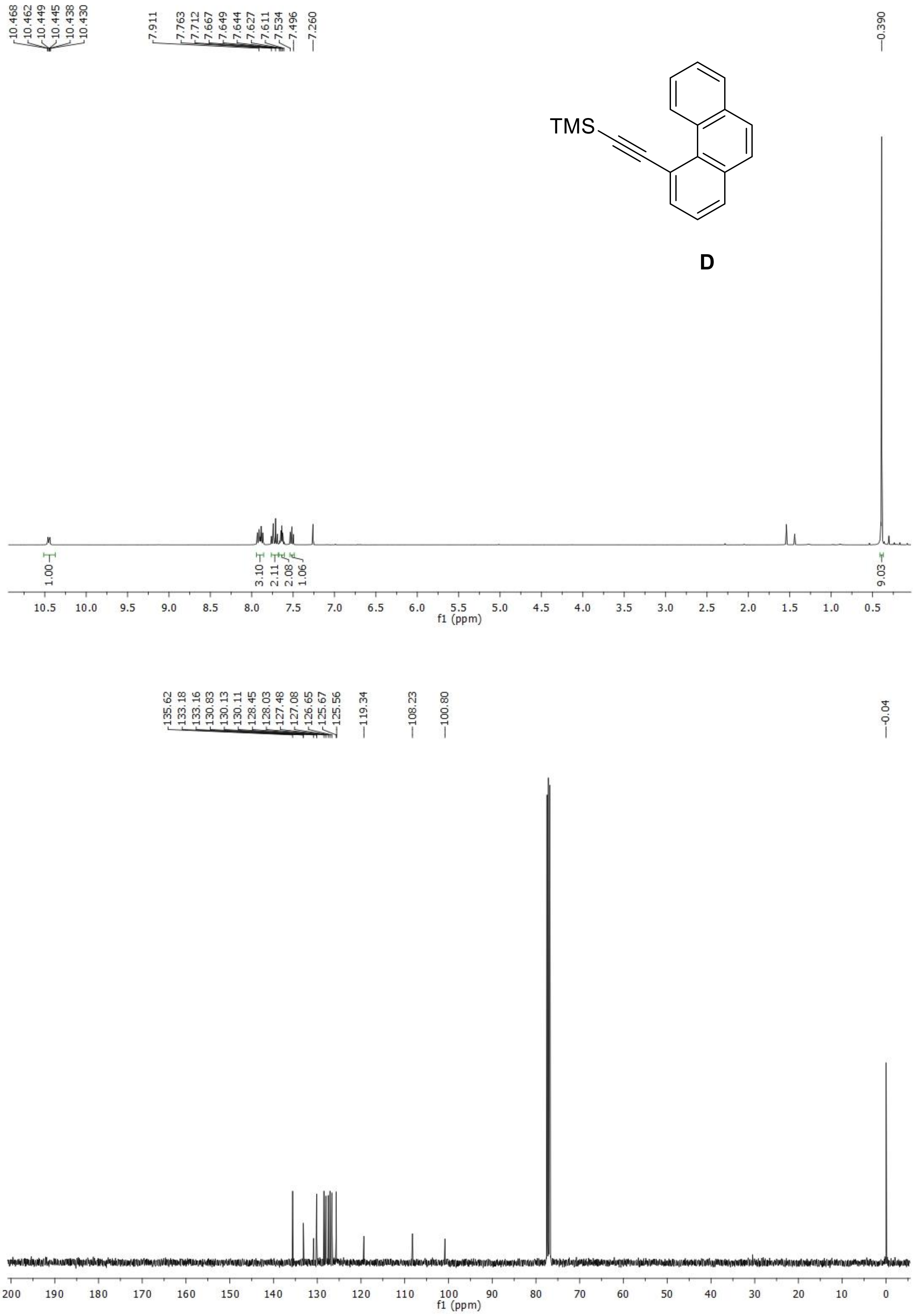

Supplementary Figure 10. ^{1}H NMR and ^{13}C NMR spectra of compound **D**.

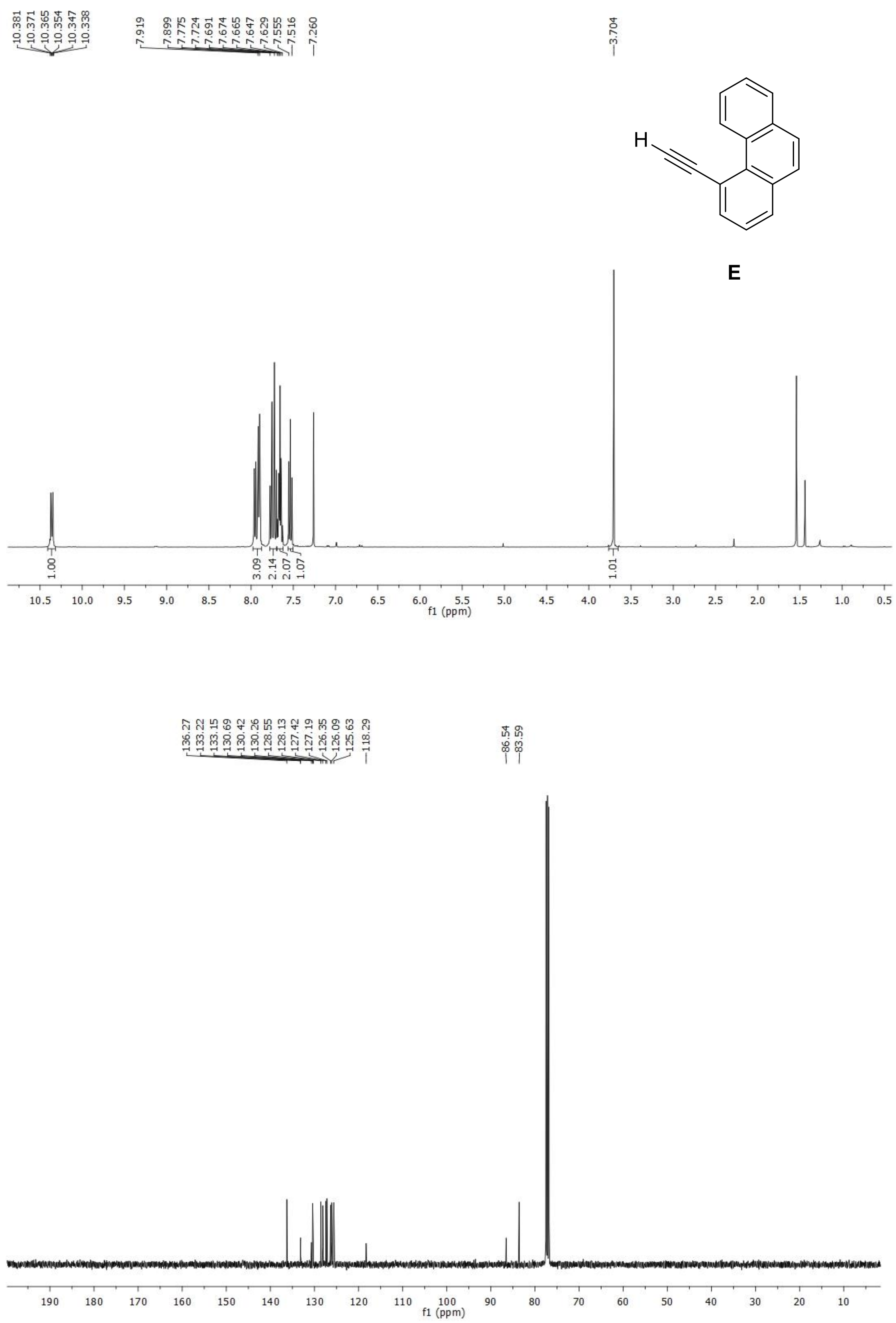

Supplementary Figure 11. ^{1}H NMR and ^{13}C NMR spectra of compound **E**.

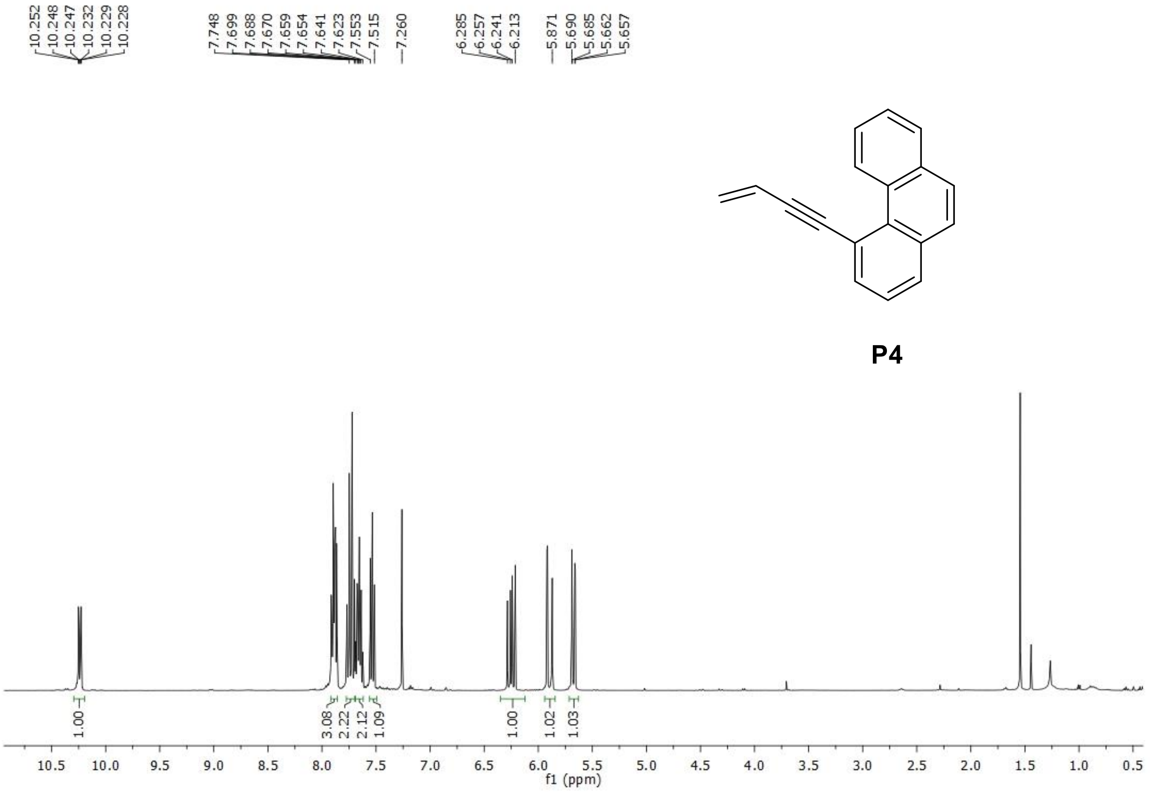

P4

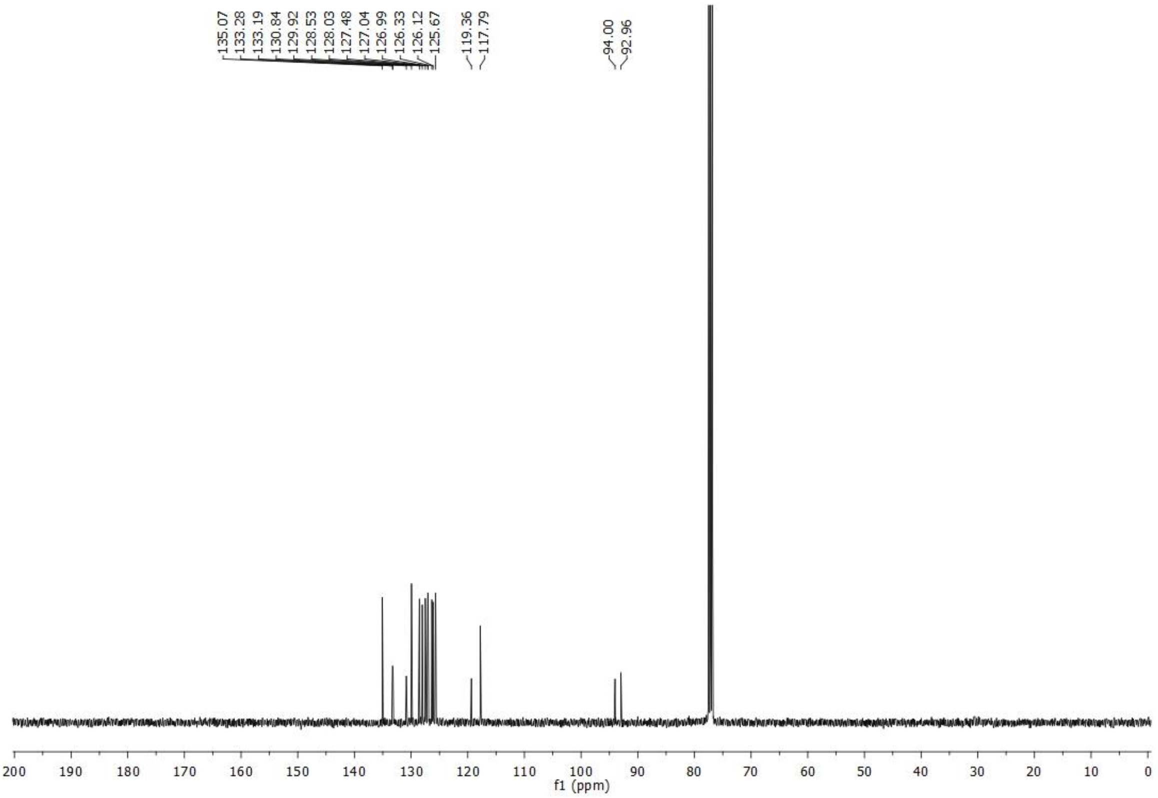

Supplementary Figure 12. ^{1}H NMR and ^{13}C NMR spectra of compound **p4**.

Supplementary References:

1 Zhao, L. *et al.* Pyrene synthesis in circumstellar envelopes and its role in the formation of 2D nanostructures. *Nat. Astron.* **2,** 413-419 (2018).

2 Schmidt, W. Photoelectron spectra of polynuclear aromatics. V. Correlations with ultraviolet absorption spectra in the catacondensed series. *J. Chem. Phys.* **66,** 828-845 (1977).

3 Johansson, K. O. *et al.* Photoionization Efficiencies of Five Polycyclic Aromatic Hydrocarbons. *J. Phys. Chem. A* **121,** 4447-4454 (2017).

4 Dewar, M. & Goodman, D. Photoelectron spectra of molecules. Part 5.—Polycyclic aromatic hydrocarbons. *J. Chem. Soc. Faraday Trans.* **68,** 1784-1788 (1972).

5 Yang, T. *et al.* HACA's heritage: A free-radical pathway to phenanthrene in circumstellar envelopes of asymptotic giant branch stars. *Angew. Chem.-Int. Edit.* **56,** 4515-4519 (2017).

6 Georgievskii, Y., Miller, J. A., Burke, M. P. & Klippenstein, S. J. Reformulation and solution of the master equation for multiple-well chemical reactions. *J. Phys. Chem. A* **117,** 12146-12154 (2013).

7 Georgievskii, Y. & Klippenstein, S. J. *Master Equation System Solver (MESS) available online at* http://tcg.cse.anl.gov/papr (2015).

8 Troe, J. Theory of thermal unimolecular reactions at low pressures. I. Solutions of the master equation. *J. Chem. Phys.* **66,** 4745-4757 (1977).

9 Mebel, A. M., Georgievskii, Y., Jasper, A. W. & Klippenstein, S. J. Temperature-and pressure-dependent rate coefficients for the HACA pathways from benzene to naphthalene. *Proc. Combust. Inst.* **36,** 919-926 (2017).

10 Wang, H. & Frenklach, M. Transport properties of polycyclic aromatic hydrocarbons for flame modeling. *Combust. Flame* **96,** 163-170 (1994).

11 Vishnyakov, A., Debenedetti, P. G. & Neimark, A. V. Statistical geometry of cavities in a metastable confined fluid. *Physical Review E: Statistical Physics, Plasmas, Fluids, and* **62,** 538-544 (2000).

12 Neimark, A. V., Ravikovitch, P. I. & Vishnyakov, A. Adsorption hysteresis in nanopores. *Physical Review E: Statistical Physics, Plasmas, Fluids, and* **62,** R1493-R1496 (2000).

13 Zhao, L. *et al.* VUV photoionization study of the formation of the simplest polycyclic aromatic hydrocarbon: naphthalene (C10H8). *J. Phys. Chem. Lett.* **9,** 2620-2626 (2018).

14 Yaws, C. Transport properties of chemicals and hydrocarbons: viscosity, thermal conductivity, and diffusivity of C1 to C100 organics and Ac to Zr inorganic. *William Andrew Inc*, ISBN:9780815520405 (2009).

15 Zador, J., Fellows, M. D. & Miller, J. A. Initiation reactions in acetylene pyrolysis. *J. Phys. Chem. A* **121,** 4203-4217 (2017).

16 Frenklach, M., Singh, R. I. & Mebel, A. M. On the low-temperature limit of HACA. *Proc. Combust. Inst.* **37,** 969-976 (2019).

17 Miller, J. A., Klippenstein, S. J. & Robertson, S. H. A theoretical analysis of the reaction between vinyl and acetylene: quantum chemistry and solution of the master equation. *J. Phys. Chem. A* **104,** 7525-7536 (2000).

18 Knyazev, V. D. & Slagle, I. R. Experimental and theoretical study of the $C_2H_3 \rightleftarrows H + C_2H_2$ reaction. Tunneling and the shape of falloff curves. *J. Phys. Chem.* **100,** 16899-16911 (1996).

19 Baulch, D. *et al.* Evaluated kinetic data for combustion modelling. *J. Phys. Chem. Ref. Data* **21,** 411-734 (1992).

20 Zagidullin, M. V. *et al.* Functional Relationships between Kinetic, Flow, and Geometrical Parameters in a High-Temperature Chemical Microreactor. *J. Phys. Chem. A* **122,** 8819-8827 (2018).

21 Harding, L. B., Georgievskii, Y. & Klippenstein, S. J. Predictive theory for hydrogen atom−hydrocarbon radical association kinetics. *J. Phys. Chem. A* **109,** 4646-4656 (2005).

22 Miller, J. A. & Klippenstein, S. J. The recombination of propargyl radicals and other reactions on a C_6H_6 potential. *J. Phys. Chem. A* **107,** 7783-7799 (2003).

23 Semenikhin, A. *et al.* Rate constants for H abstraction from benzo(a)pyrene and chrysene: a theoretical study. *Phys. Chem. Chem. Phys.* **19,** 25401-25413 (2017).

24 Mebel, A. M., Landera, A. & Kaiser, R. I. Formation mechanisms of naphthalene and indene: from the interstellar medium to combustion flames. *J. Phys. Chem. A* **121,** 901-926 (2017).

25 Brown, H. C., Hamaoka, T. & Ravindran, N. Reaction of alkenylboronic acids with iodine under the influence of base. Simple procedure for the stereospecific conversion of terminal alkynes into trans-1-alkenyl iodides via hydroboration. *J. Am. Chem. Soc.* **95,** 5786-5788 (1973).
